# Optical Microneedle–Lens Array for Selective Photothermolysis

**DOI:** 10.3390/mi15060725

**Published:** 2024-05-30

**Authors:** Jongho Park, Kotaro Shobayashi, Beomjoon Kim

**Affiliations:** 1 Institute of Industrial Science, The University of Tokyo, Tokyo 153-8505, Japan; johopark@iis.u-tokyo.ac.jp; 2 Department of Precision Engineering, School of Engineering, The University of Tokyo, Tokyo 113-8656, Japan; kotaro-s@iis.u-tokyo.ac.jp

**Keywords:** microneedle, lens array, optical, laser therapy, selective, localized light delivery, photothermolysis

## Abstract

Photothermolysis is the process that converts radiation energy into thermal energy, which results in the destruction of surrounding tissues or cells through thermal diffusion. Laser therapy that is based on photothermolysis has been a widely used treatment for various skin diseases such as skin cancers and port-wine stains. It offers several benefits such as non-invasiveness and selective treatment. However, the use of light, e.g., laser, for safe and effective photothermolysis becomes challenging due to the limited penetration of light into skin tissue as well as the presence of melanin, which absorbs this light. To solve the current issues, we propose an optical microneedle–lens array (OMLA) coated with gold in this work to directly deliver light to targeted skin layers without being absorbed by surrounding tissues as well as melanin, which results in the improvement of the efficiency of photothermal therapy. We developed a novel fabrication method, frame-guided micromolding, to prepare the OMLA by assembling two negative molds with simultaneous alignment. In addition, evaluations of the optical and heat transfer characteristics of the OMLA were performed. We expect our developed OMLA to play a crucial role in realizing more effective laser therapy by allowing the precise delivery of photons to the target area.

## 1. Introduction

Light therapy is a non-invasive procedure that involves exposing the skin to light with specific wavelengths to treat skin conditions like scars, acne, and vascular lesions. There are various types of light therapy available today, which use a wide range of wavelengths, from visible to non-visible ultraviolet light. Some of the common types include laser therapy, intense pulsed light therapy (IPL), low-level laser therapy (LLLP), and photodynamic therapy (PDT).

Since L. Goldman reported the effect of a laser beam on human skin to treat lesions of pigmented skin areas [1], laser therapy has been applied to treat hemangiomas [2], the dilation of blood vessels [3], and other skin-related diseases, such as dyschromia [4] and acne [5]. Similarly, IPL, based on the use of a wide-ranging spectrum from 500 nm to 1300 nm, was first used to treat hemangiomas by Mühlbauer et al. in 1976 [6]. The wavelength band could be controlled using filters, and IPL treatment has a high skin coverage rate [7].

LLLT (low-level laser therapy) uses lower energy and is based on the absorption of light energy by the cells, which causes the photochemical reaction leading to ATP reduction, improved blood flow, and increased reactive oxygen [8,9]. PDT is an established method to treat skin disorders such as skin tumors, proliferative skin disease, as well as precancerous diseases. It involves the administration of a photosensitizer followed by its activation by a light source to generate cytotoxic reactive oxygen species (ROS) [10,11]. Singlet oxygen is the primary cytotoxic agent, and maximizing its generation is crucial for increasing PDT’s therapeutic efficacy [12].

The mechanism of laser therapy is based on selective photothermolysis [13,14]. Photothermolysis is a process that converts radiant energy into thermal energy, which leads to the destruction of vascular tissues through thermal diffusion. The process of selective photothermolysis can be broken down into three phenomena: the conversion of light into heat, heat transfer, and denaturation. By using a laser to irradiate the skin’s surface at a wavelength that is well-absorbed by the target hemoglobin (in the ranges of 400~450 nm and 510~590 nm), temperatures that are higher than 70 °C can be generated in the target area, resulting in the destruction of the target [15,16].

Although laser therapy has been researched and developed for the treatment of various skin diseases, there exist several challenges. The first challenge is the penetration of light into the skin. Light in human skin can only travel a limited distance due to scattering and absorption caused by different tissue components, such as cells, organelles, and various fiber structures. A simulation performed by Maeda et al. showed that the depth of light penetration is influenced by the wavelength of the light source [17]. For example, light with a wavelength below 600 nm can only penetrate 0 to 1 mm, while light over 600 nm in wavelength can reach a maximum depth of 5 mm. This limitation of light therapy is well-illustrated by the fact that when the depth of a lesion exceeds 1030 μm, the results are generally poor compared with superficial lesions below 830 μm [18].

The second challenge is the presence of melanin in the skin. Melanin is a pigment that is the primary component of the epidermis and shields the skin from ultraviolet rays. It has been known that the absorption coefficient decreases as the wavelength increases [19]. In addition, melanin has a wide absorption range that starts from UV to the infrared region and overlaps with hemoglobin’s absorption band. As a result, the treatment efficiency of photothermolysis can be reduced or limited as photons are absorbed by the melanin in the epidermis layer before reaching the final target, hemoglobin.

Melanin not only restricts light’s penetration inside the skin but also generates heat due to its high absorption coefficient, resulting in thermal injury. When the thermal damage in the skin exceeds the threshold value, it can cause necrosis, vascular thrombosis, and a decrease in dermal collagen birefringence [20]. Overall, the epidermis layer, which contains various biological components such as melanin, which absorbs and scatters light, poses a significant challenge for light therapy. Thus, it is necessary to develop new therapeutic methods in laser therapy to solve the issues described above, which would result in the optimized treatment of skin diseases.

Recently, microneedle array patches (MAPs) have attracted much attention as an alternative to conventional syringes and administration methods. Several types of MAPs have been researched and developed for biomedical applications such as drug delivery and medical treatment. The types of MAPs include solid, coated, dissolvable, porous, and hydrogel types [21]. Each type has different characteristics and functions. Among them, solid-type MAPs can be used to enhance light penetration as a light guide, making them an advantageous tool for light therapy. For example, Kang et al. proposed light-guiding microneedles, where light penetrates the skin by undergoing total internal reflection [22]. The microneedles were integrated with LED lights to guide thermal energy from the light source, causing photocoagulation and the occlusion of the target. Internal reflection was induced inside the microneedle structures, accumulating light at their tips. They demonstrated that light transmission can be enhanced more than 1.6 times with microneedles compared with light transmission with only LEDs. However, there are still some questions regarding the light transmission efficiency that need to be addressed.

Meanwhile, Kono et al. proposed an optical microneedle–lens array (OMLA) that combines a microneedle array and a lens array to concentrate light with a wide incident angle [23]. In addition, Wu et al. demonstrated the feasibility of an OMLA as a tool for the localized delivery of blue light into the skin layer for the treatment of melanoma [24]. However, OMLAs have a critical drawback in that the microneedles and lenses are fabricated separately and prepared by combining the two parts without precise alignment. As a result, several issues have been confirmed, such as light scattering and the subsequent decrease in light transmission efficiency.

**Figure 1:**
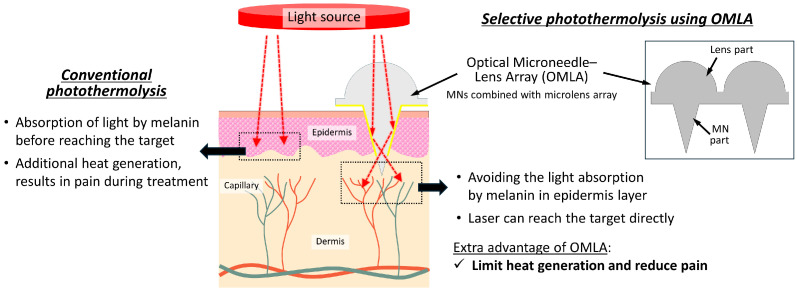
Schematic diagram of proposed OMLA for selective photothermolysis.

In this work, we propose a new fabrication process for an optical microneedle–lens array (OMLA) that has a lens array aligned with the respective microneedles (MNs) in a unit patch, which reduces the fabrication time and improves the alignment resolution (Figure 1). To realize the unit fabrication of the OMLA, we introduced a frame-guided micromolding technique that includes assembling two negative molds in a metal frame, MNs, and a lens array, which are aligned simultaneously. In addition, we designed the geometry of the lens and microneedles using the finite element simulation to ensure that the focal length is located near the tip, allowing light to accumulate at the tip. Thus, the transmission of light to the epidermis layer, where melanin exists, can be minimized by preventing the melanin from absorbing light, which results in maximizing the light transmission efficiency. Moreover, we demonstrated the coating of the microneedle tips using gold sputtering to enable total reflection inside the microneedles regardless of the light’s incident angle.

## 2. OMLA Design Principle

Regarding the design of the OMLA in this work, we considered a port-wine stain (PWS) as a representative target for selective photothermolysis using OMLA. A PWS is a vascular lesion characterized by the dilation of capillary vessels located in the papillary and mid-reticular layers of the dermis. It is treated by the laser irradiation of the affected area in terms of selective photothermolysis to induce the photocoagulation of dilated vessels [25].

**Figure 2:**
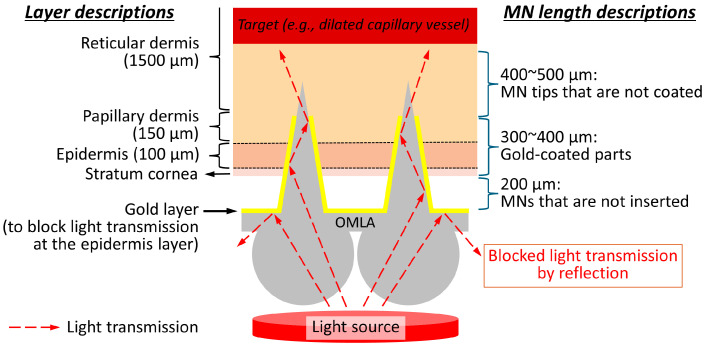
Details of design principle of OMLA.

Assuming the tip of the OMLA reaches around the middle part of the reticular layer, we determined the total length of the microneedles as 900 μm, supposing that the thicknesses of the stratum cornea, epidermis, papillary dermis, and reticular dermis are 10, 100, 150, and 1500 μm, respectively (Figure 2). In addition, we added 200 μm as the extra part that is not inserted, considering the influence of skin elasticity during skin penetration [26]. The length of the side at the bottom was determined as 300 μm, which results in a vertex angle of 18.92°.

In addition, we introduced a coating of the microneedles’ surfaces using gold metal, except the tips of the microneedles, so that the light is accumulated at the tips and then transmitted through the skin layer without being absorbed by the epidermis as well as the stratum cornea. We chose gold as a coating material considering its biocompatibility [27], reflectance [28], and availability as well as processability for the coating process. Herein, we expected the length of the coated part to be from 400 to 500 μm.

For the lens array, we set the diameter of each lens as 1.5 mm considering the MNs’ dimensions and array as well as the handling during the fabrication process. As a result, the final distance between two adjacent tips of MNs was designed to be 1.5 mm so that the apexes of the MNs are positioned at the centers of the lenses.

## 3. Materials and Methods

### 3.1. Fabrication of Optical Microneedle–Lens Array (OMLA)

We used micromolding and hot press methods to fabricate the OMLA in this work. First, we fabricated two different negative molds for the MNs and the lens array, respectively. Also, we fabricated and used an exterior frame to make two negative molds for the MNs and the lens array to be aligned and fixed during the PLA casting process.

**Figure 3:**
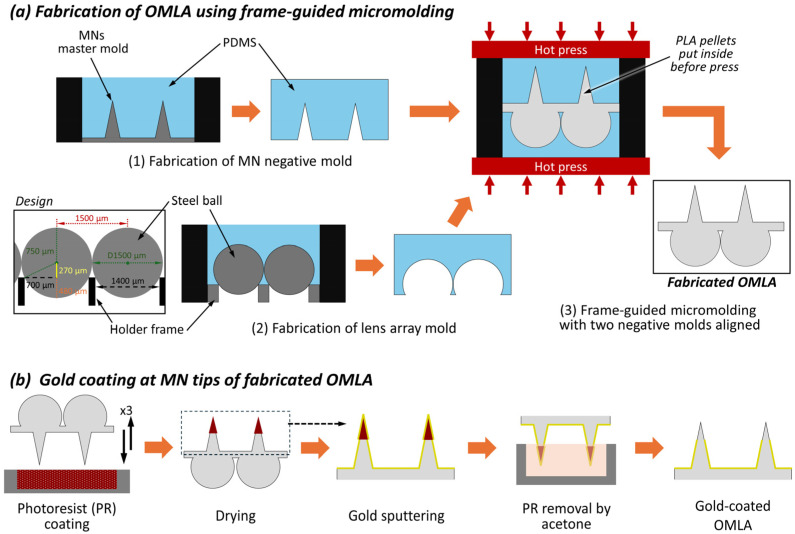
Fabrication process for OMLA: (**a**) frame-guided micromolding process for unified OMLA fabrication; (**b**) gold-coating process using a modified lift-off method.

For the master mold for the MN array, we used wire electrical discharge machining as the fabrication method and a polished tungsten carbide plate as the substrate. The MNs were designed with a square pyramidal shape considering the machining process. The length and + side of the square MN base were designed to be 900 and 300 μm, respectively, as described above, and were arrayed in a square layout of 15 × 9. The center-to-center distances in the horizontal and vertical directions were set to 750 and 1300 μm, respectively (Figure 3a(1)).

For the master mold for the lens array, we prepared a metal holder to place steel balls (SBM-SUJ-1.5, Misumi Group Inc., Tokyo, Japan) for the fabrication of negative molds using a conventional precision-machining process. The holder was a square plate that had an exterior dimension of 20 × 20 × 1 mm and a 7(8) × 9 array of holes whose diameter was 1400 μm. Here, we designed and fabricated the holder frame so that it can hold steel balls whose diameter is 1.5 mm in an array with a tightly packed layout. As a result, the steel balls are exposed at a height of 1020 μm above the surface of the holder (Figure 3a(2), design inset).

Next, we fabricated negative molds for the MNs and lens array for the micromolding process. To make the thickness as well as the width of the negative molds consistent, we fabricated and used a cylinder-shaped hollow frame that had a height of 10 mm and a length of 20 mm inside. A mixture of polydimethylsiloxane (PDMS) prepolymer and a curing agent (10:1, *w*/*w*, DOWSIL™ SILPOT 184 W/C, Dow Chemical Company, Midland, MI, USA) was poured in the master mold, defoamed at 20 kPa for 1 h, and cured in an oven at 90 °C for 1 h in both cases. The cured negative mold was released from the master mold and subsequently used for the fabrication of the OMLA.

For the OMLA micromolding with alignment, we prepared and used a square cylinder that had an inner side of 20.2 mm and a height of 20 mm to make both molds fit inside with their alignment (Figure 3a(3)). We used polylactic acid pellets (3D850, Nature works LLC, Plymouth, MN, USA) as the material for the OMLA. After sealing the bottom side of the cylinder frame using Kapton^®^ tape (DuPont de Nemours Inc., Wilmington, DE, USA), the negative mold of the lens array was placed and attached to the bottom of the cylinder frame. The cylinder frame with the lens array mold was set on the plate of a small heat-press machine (H300-01, AS ONE Co., Osaka, Japan). PLA pellets of 0.55 g were set on the center of the lens array mold and heated up to 210 °C to completely melt the PLA pellets. Once all pellets were melted, the negative mold for the MNs was positioned onto the lens negative mold. The mold set was then pressed at 1 MPa for 10 min while maintaining the temperature and pressure. Then, the heaters were turned off, and the mold was cooled down in the ambient air for 1 h. Finally, the heat-pressed mold set was removed from the heat press and disassembled to retrieve the OMLA.

### 3.2. Coating of Gold Layer onto the Fabricated OMLA

We used a modified lift-off method to coat the tip of the OMLA only in this work (Figure 3b). First, the positive photoresist (S1818, Dow Chemical Company, Midland, MI, USA) was soaked into a nonwoven fabric wipe and put inside a reservoir tray. A four-axis stage controller (Mark-204AM-MS, Sigma Koki Co., Ltd., Saitama, Japan) and a motorized stage (SGAM(MS)26-100(Z), Sigma Koki Co., Ltd., Saitama, Japan) were used to precisely move the OMLA during the coating process. In addition, the OMLA samples were monitored during the whole coating process using a digital microscope (Dino-Lite, AD7013MTL, AnMo Electronics Co., Hsinchu, Taiwan).

After the OMLA was fixed onto the plate of the stage upside down, the OMLA was moved downward until it touched the top of the photoresist-soaked wipe. From the contact point, we moved 200 μm downward using the controller to dip the tip parts into the wipe and limit the coated height. After repeating the coating process 3 times, the OMLA was dried for 10 min to evaporate the organic solvent from the photoresist.

Second, the photoresist-coated OMLA was placed in the chamber of a gold coater (SC-701MkII, Sanyu Electron Co., Ltd., Tokyo, Japan). Gold coating was then performed by sputtering using a gold target (ϕ49 mm; t0.05 mm) under 30 Pa of vacuum pressure. The electric current was set to 5 mA, and the deposition time was adjusted to achieve a specific gold layer thickness. The MN parts of the gold-coated OMLA were then submerged into acetone. After dipping for 10 min, the OMLA was cleaned with ethanol and deionized water. Finally, the OMLA was fully dried in a desiccator and stored until its use. The fabricated OMLA was observed using digital microscopes (VH-5500, Keyence Co., Osaka, JAPAN/Stemi 305 with Axiocam 105 color, Carl Zeiss AG, Oberkochen, Germany).

### 3.3. Optical Evaluation of Fabricated OMLA

For the optical evaluation of the fabricated OMLA, we evaluated the intensity of transmitted light through the OMLA while being inserted into an artificial skin model. We used a laser module with a wavelength of 650 nm (VLM-650-03-LPT, Quarton Inc., New Taipei City, Taiwan) with an X-Y-R precision stage to adjust the incident angle of the laser module and took microscopic images of the OMLA using a digital microscope (UM12, MicroLinks Technology Corp., Kaohsiung, Taiwan). As a skin model, we prepared a gelatin block to mimic the dermis layer. Briefly, gelatin powder from porcine skin (Gelatin Type A, from Porcine Skin, Nitta Gelatin Inc., Osaka, Japan) was mixed in DI water at a ratio of 1:10. The mixture was then agitated at 300 rpm and heated up to 60 °C for 1 h for complete dissolution. The mixture was then poured into a glass dish and cooled at room temperature. The block was finally cut into a specific size and used for experiments.

For the preparation of the OMLA experiments with the gelatin block, aluminum foil was first attached to the MN side of the OMLA to block the unexpected transmission or diffraction of light from the lens side. The OMLA was then penetrated into the skin model and set on a sample holder that had a digital microscope horizontally facing the MN tips of the OMLA. Image J was used to calculate the grey value plotted with the relationship between the incident angle of the laser and the light intensity.

### 3.4. Evaluation of Gold-Coated OMLA

The evaluation regarding light irradiation with the gold-coated OMLA was performed with the same experimental setup described in the previous section. In addition, we used a fluorescent dye solution, 0.15% (*w*/*v*) of Rhodamine B (R6626, Merck KGaA, Darmstadt, Germany), mixed with gelatin mixture to visualize the light irradiation at the MNs inside the gelatin block. At the same time, the laser module with a wavelength of 520 nm (VLM-520-52-LPT, Quarton Inc., New Taipei City, Taiwan) was used to excite the rhodamine B fluorescent dye. Fluorescent images were obtained using an inverted fluorescent microscope (IX71, Olympus Co., Tokyo, Japan).

To measure the power transmitted by the gold-coated OMLA, we used an integrating sphere photodiode power sensor (S142C, 350~1100 nm, 1 µW~5 W, Thorlabs, Inc., Newton, NJ, USA) and a power meter console (PM100D, Thorlabs, Inc., Newton, NJ, USA) in the setup stage described above. We prepared MAPs with MNs only, the OMLA, and the gold-coated OMLA and inserted them inside the gelatin skin model to evaluate the power transmission efficiency.

To evaluate the heating behavior of the gold-coated OMLA, we chose a YAG laser with a wavelength of 1064 nm (MIL-N-1064, CNI Optoelectronics Tech. Co., Ltd., Changchun, China) and irradiated it at 150 mW. Infrared digital thermography (FSV-GX7700, Apiste Corporation, Osaka, Japan) was used to monitor the thermal behavior of the OMLA from the top of the OMLA. Here, a porcine skin (Funakoshi Co. Ltd., Tokyo, Japan) was used as the skin model. The porcine skin was stored in a freezer at −20 °C before its use and used for the evaluation after it was taken out and kept at room temperature for 1 h. The gold-coated OMLA was then inserted into the skin specimen, and temperature changes in the OMLA were recorded by taking thermographic images every 1 s at a 2 cm distance from the side of the OMLA.

## 4. Results and Discussion

### 4.1. Fabrication of Optical Microneedle–Lens Array (OMLA)

**Figure 4:**
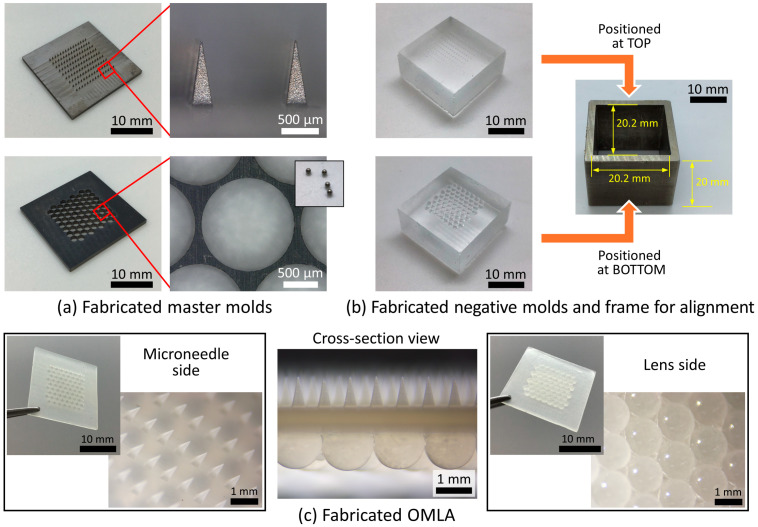
Fabrication results of OMLA: (**a**) fabricated metal master molds for MN array and lens array, (**b**) negative PDMS molds fabricated from respective master molds and square cylindrical frame for molds assembly, and (**c**) overview of fabricated OMLA; from left: MN side, side view, and lens side.

The fabricated master molds for the MN array and lens array are shown in Figure 4a. The length and the side of the square MN base were 900 ± 15 and 306 ± 7 μm, respectively (N = 10). The center-to-center distances were 755 ± 2 μm horizontally and 1311 ± 2 μm vertically (N = 10). 

The square-shaped holder for the lens array had a length of 20.05 mm and circular voids whose diameter was around 1400 μm for positioning the steel balls (diam.: 1.5 mm, inset) in a well-packed manner. After the fabrication of both master molds, negative PDMS molds were fabricated. Here, we used a square hollow cylinder that had a height of 10 mm and a length of 20 mm inside the cylinder to limit the height and the exterior dimension of the respective negative PDMS molds. In addition, for the lens array, we positioned all steel balls onto the cavities of the holder and performed PDMS filling and curing processes.

Figure 4b shows the fabricated negative PDMS molds. Both molds had a height of 9.6 mm and a length of 19.8 mm on one side. We confirmed that the decrease in the dimension of the negative molds was caused by the shrinkage of the PDMS during the curing process. Similar to the fabrication of the negative PDMS molds, we used a square cylinder whose height was 20 mm to prevent the movement of the PDMS molds during the heat-press process. In addition, we designed the inside length to be 20.2 mm so that the mold had margins for positioning both molds inside and following assembly for the OMLA’s fabrication (Figure 4b, square cylinder).

The fabricated OMLA is shown in Figure 4c. On both sides, we confirmed that the microneedle parts and lens arrays were successfully fabricated using the frame-guided micromolding method. The length and base of each MN were 902 ± 6 and 313 ± 6 μm, respectively. The thickness of the base layer of a fabricated OMLA patch was 889 ± 24 μm (N = 12). We confirmed that the thickness of the base was fabricated close to the optimal thickness from the results of computational simulations (Supplementary Materials). In addition, it well matched the calculation for the thickness design of the square cylinder frame. The diameter of the lens and the height of the lens exposed above the base were 1455 ± 8 μm (N = 18) and 1025 ± 4 μm (N = 17), respectively. Although the diameter of the fabricated lens was slightly decreased compared with that of steel balls, the height of the exposed lens part, around 1024 μm, was fabricated as close to the designed value (1020 μm, Figure 3a(2)). Regarding the frame-guided micromolding mechanism that controlled the whole thickness of the OMLA by the height of the exterior walls, we considered several dimensions that could be affected by one another. Here, the thickness of the negative PDMS molds as well as the height of the square cylinder for assembly can affect the length or thickness of the fabricated microneedles, lenses, and base layers. Thus, fixing the specific dimensions of each component or introducing additional components can help to fabricate the OMLA with more precisely controlled dimensions.

### 4.2. Optical Analyses of Fabricated OMLA without Gold Coating

#### 4.2.1. Evaluation of Light Intensity with Different Incident Angles

To investigate the light transmission with our fabricated OMLA, we irradiated the light using a laser module with a wavelength of 650 nm and evaluated the light intensity by image analysis. We used a gelatin block to mimic the dermis layer as it has high transparency for optical evaluation and has a refractive index that is close to the dermis layer [29].

**Figure 5:**
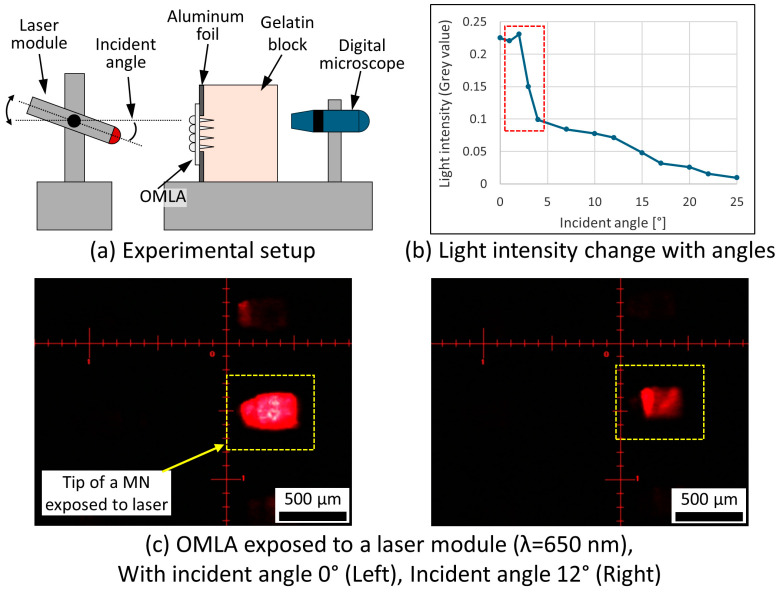
Light intensity evaluation while changing the incident angle: (**a**) schematic diagram of experimental setup, (**b**) measurement results with respect to different incident angles, and (**c**) microscopic images with incident angles of 0° (**left**) and 12° (**right**).

The experimental setup is shown in Figure 5a. The images of the tip of MN were taken while changing the incident angle of the laser module by using the X-Y-R precision stage. The measured light intensity was acquired by using grey values and plotted as a function of the incident angle of the laser (Figure 5b). Figure 5c shows the representative images of the MN tips with incident angles of 0° and 12°, respectively. We observed a drop in the light intensity when the incident angle exceeded 3° (red rectangle, Figure 5b), whereas the simulation results showed a gradual drop from 0° (Supplementary Materials). Regarding the difference compared with the simulation, we considered that the optical axis of the laser module was not precisely aligned with the center of the target lens on the OMLA at 0°, the starting point of measurement, as we used a precision stage to control the angles manually.

From the results, we suggest that the light source should be positioned nearly perpendicular to the fabricated OMLA to achieve maximum light transmission. In addition, it was considered that the surface roughness as well as the dimension differences in the lens array in the OMLA caused the difference in the intensity measurements. Thus, we consider that finer control in the fabrication process will help to achieve higher light transmission.

#### 4.2.2. Evaluation of Misalignment between MNs and Lens Array

Due to our fabrication method, frame-guided micromolding, it was that expected the misalignment between the MNs and lens array would occur during the molding process. Thus, we evaluated the influence of misalignment using a fabricated OMLA before gold coating.

**Figure 6:**
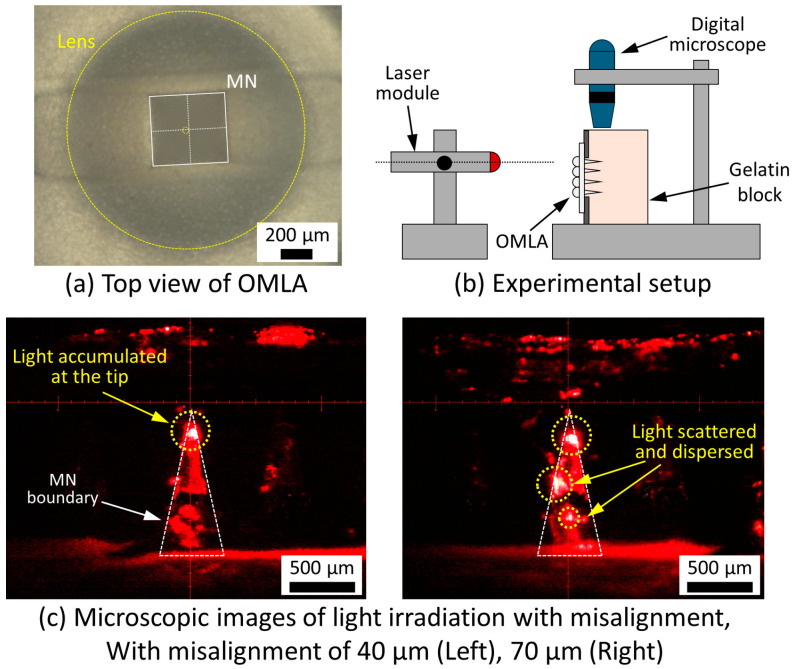
Evaluation of center misalignment in fabricated OMLA: (**a**) experimental setup, (**b**) representative image of misalignment of an MN and a lens, and (**c**) light irradiation image with two different misalignments.

First, we took microscopic images of the OMLA with white light from the top side. We then measured the distance between the center of the MN and the center of the corresponding lens on a different side. Figure 6a is the representative image showing the misaligned state of the MN part (white lines and rectangle) and lens part (yellow ones). The average distance of center misalignment was 52 ± 23 μm (N = 6) with our fabricated OMLA. Comparing the dimensions of the fabricated PDMS molds and the frame, we considered that the OMLA could achieve around 80% efficiency of photon transmission based on our computational simulation results.

Next, we evaluated the light transmission with the misalignment using an experimental setup that was similar to the previous evaluation described above. In this case, we positioned the microscope on the top side of the OMLA (Figure 6b). Figure 6c shows microscopic images of the OMLA with light irradiation from the top view. It was confirmed that the light accumulated well at the tip of the MN in the case of a misalignment distance of 40 μm. In this case, we expected that our device would meet the requirement of light transport targeting the epidermis and dermis layer without scattering inside. On the contrary, we also confirmed that some light was scattered inside the MN body with 70 μm of misalignment (Figure 6c, right). We confirmed that the results showed a similar tendency that we achieved with computational simulations (Figure S6). We suppose that the light scattering was caused by the shift in the focal length originating from the misalignment of the MN and lens centers. As the light scattering inside the OMLA may cause unexpected results, such as damaging surrounding cells with following pain in practical use, it was considered that more precise alignment is necessary during the OMLA fabrication process.

### 4.3. Optical Evaluation of Gold-Coated OMLA

#### 4.3.1. Gold Coating on OMLA

**Figure 7:**
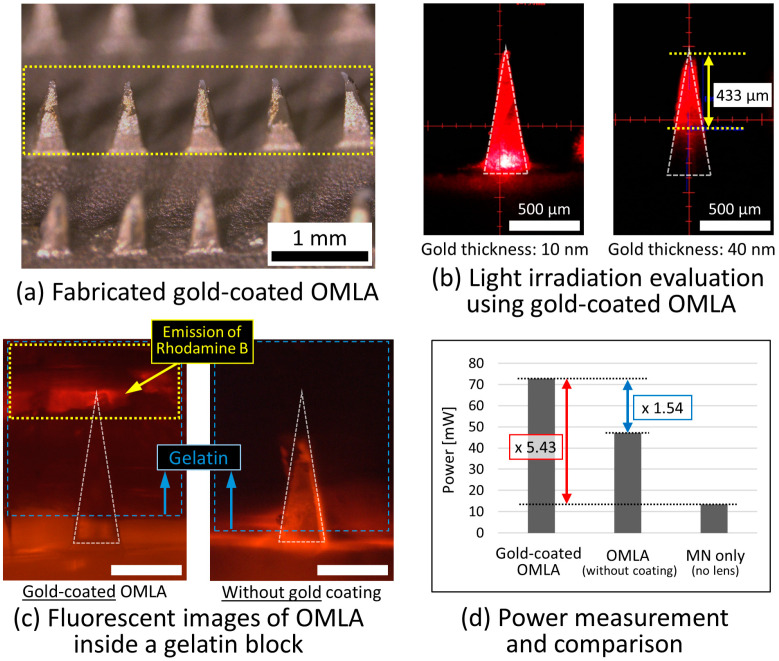
Gold-coated OMLA and evaluation results: (**a**) Fabricated gold-coated OMLA, (**b**) light irradiation evaluation with respect to different gold thicknesses, (**c**) fluorescent image of OMLA and gold-coated OMLA inside a gelatin block (scale bar: 500 μm), and (**d**) power measurement results using a photodiode power sensor.

Using the modified lift-off method, we coated a gold layer onto the fabricated OMLA except the tip (Figure 7a, yellow rectangle). We used a photoresist-soaked wipe as the reservoir in this work to prevent the photoresist’s moving upward due to its surface tension. After the coating and drying process, gold was coated over the whole surface by sputtering and was removed from only the tip parts with acetone. After the lift-off process, we observed the loss of the gold coating on some parts (Figure 7a, second MN from the right). At the same time, it was observed that too short or too long a coating process could cause the peeling off or the failure of removal during the lift-off process. We considered that insufficient adhesion or the stiction of gold onto the PLA material due to the generated heat during the gold-coating process caused such results, respectively. Finally, we achieved, overall, 70% of the successful rate for the gold coating onto the OMLA with a thickness of 40 nm.

#### 4.3.2. Evaluation of Light Irradiation Using Gold-Coated OMLA

Next, we investigated the light irradiation changes with different thicknesses of the coated gold layer. The experimental setup was the same as that in the previous section (Figure 7b). The evaluation was performed using gold-coated OMLAs with three different thicknesses: 10 nm, 40 nm, and 60 nm. Figure 7b shows representative images of the gold-coated OMLAs with thicknesses of 10 and 40 nm. The results show that the gold thickness of 10 nm could not block the transmission of light, which well matches the results of a previous study, where the reflectivity of a gold film reached a plateau at over 50 nm [28]. In the results with a thickness of 40 nm, we observed that the light accumulated at the tip of the microneedle (Figure 7b, right). It is important to note that the thickness of 40 nm of gold was sufficient to block the light transmission except for in the exposed part. Here, the length of the exposed tip part where light was accumulated was around 430 μm. The OMLA coated with 60 nm thick gold showed a similar result to that with 40 nm; however, we finally chose a 40 nm thick coating to prevent the stiction of the gold layer caused by heat from a long deposition time.

To investigate the light that exits from the MN tip and its influence around the tip, we performed fluorescent imaging using the same experimental setup as in the previous section. In addition, we added rhodamine B dye into the gelatin block and excited the rhodamine B through the OMLA using the laser module with a wavelength of 520 nm. Here, we speculated that the irradiation of light into the gold-coated OMLA would result in fluorescence near the tip of the microneedle only. In the results (Figure 7c), we clearly confirmed the fluorescence at the tip of the gold-coated OMLA and differentiated the light response between the gold-coated OMLA and a pristine OMLA. For the gold-coated OMLA, the top part of the gelatin was illuminated with a yellow–orange color that corresponded to emission at a 580 nm wavelength. At the same time, it was observed that the emission was spread in the horizontal direction mainly. As the gelatin block was used as a skin model here, it was considered that different optical characteristics of the gelatin contributed to the results that were different from the simulation results (Figure S2 or Figure S3b). Meanwhile, the gelatin with the pristine OMLA showed overall fluorescent emission along the inserted MN structure as the laser light spread through the MN, which resulted in the emission of rhodamine B around the MN structure.

#### 4.3.3. Evaluation of Power Transmission of Gold-Coated OMLA

For the quantitative analysis of the gold-coated OMLA, we evaluated the power transmission using an integrating photodiode power sensor. In addition, we used the gold-coated OMLA, an OMLA without a coating, and a microneedle array without a lens. All microneedle array samples were prepared by inserting them into a gelatin block. In addition, aluminum foil covered the surface of the OMLA without a coating as well as the MN array, except for the MN structures, to make the experimental conditions the same. The output power for a laser module was set to 500 mW, and the power of light transmission was measured.

Figure 7d shows the measurement results. The results show that there was a 5.43-fold increase for the gold-coated OMLA compared with the single microneedle without a lens (red rectangle text) and a 1.54-fold increase compared with the OMLA without a gold coating (blue rectangle text). Thus, we consider that the light from the source was accumulated at the tip part successfully with the fabricated gold-coated OMLA, which resulted in the functional design of the OMLA being realized as expected.

### 4.4. Evaluation of Heating Behavior of Gold-Coated OMLA

As the developed OMLA was designed to treat port-wine stains based on selective photothermolysis in this work, we evaluated the heat transfer with temperature changes when the OMLA, which was inserted into porcine skin, was irradiated with an infrared laser. Here, porcine skin was chosen as the skin model due to its similar anatomy, physiology, and transdermal absorption characteristics to human skin [30].

**Figure 8:**
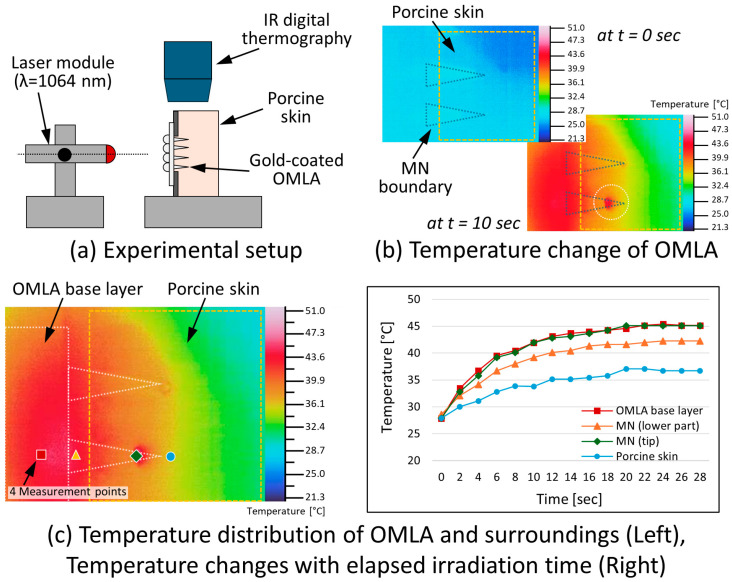
Heating behavior of gold-coated OMLA: (**a**) Experimental setup to measure temperature changes, (**b**) temperature changes in OMLA at 0 and 10 s of irradiation, (**c**) temperature distribution of OMLA and surroundings at 10 s (**left**) and temperature changes in each part with respect to elapsed irradiation time (**right**).

Figure 8a shows the experimental setup that included an IR laser module with a wavelength of 1064 nm. At the beginning of irradiation, the temperature was around 28 °C on the interface between the MN tips and the porcine skin (Figure 8b, left top). In addition, we confirmed that the temperature reached around 42 °C at the MN tips after 10 s of irradiation (Figure 8b, white circle). Here, we also observed that a temperature increase did not occur at the tip of the MN that is shown on the upper side. We considered that the incomplete removal of the gold layer on the tip caused such a result. At the same time, an increase in temperature was confirmed on the OMLA itself as well as the porcine skin.

Figure 8c shows the temperature distribution after 10 s of laser irradiation. We measured the temperatures at four different points and plotted the values with respect to the elapsed irradiation time (Figure 8c, right; the measurement points and markers were drawn as matched). In the results, it was clearly observed that the skin temperature was around 34 °C, whereas the temperature of the OMLA base layer reached around 42 °C at 10 s. In addition, the maximum temperature was 45.1 °C (MN tips) and 37.0 °C in the porcine skin. Although the light accumulated at the tip of the gold-coated OMLA successfully, we observed that the temperature of the OMLA parts as a whole increased altogether.

Here, we confirmed that the temperature of the MN tips had the same tendency and ranges as that of the base layer at the highest temperature values. At the same time, the lower part of the OMLA had a lower temperature than the other parts of the OMLA. Regarding the temperature increase at the base layer, it was considered that the light was concentrated inside the base layer due to the lens structures, which caused the highest temperature among all measured points. The lower parts of the MNs were directly connected to the base layer, but they were exposed to ambient air. As all parts of the OMLA are made of the same material, PLA, it was expected that the temperature of the lower parts would have similar or lower values depending on the penetrated portion.

Meanwhile, the temperature of the penetrated parts was slightly lower. It was considered that the gold coating caused the blockage of light spreading outward with reflection inside. Finally, the tip part had as high a temperature as the base layer, which showed that the incident light was transmitted and exited from the exposed tip part.

The temperature of the porcine skin was the lowest among the measurement points. We considered that the light dissipated and spread right after its exit from the tip parts. It was also confirmed that the temperature was not sufficient for the selective photothermolysis effect near the MN tips. Thus, we tried to increase the laser power from 150 to 500 mW. With increasing power, we could obtain a higher temperature of around 43 °C in the skin; however, the temperature of the OMLA base layer increased up to 50 °C at the same time, which can cause unexpected thermal damage. Yet, it was confirmed that the temperature at the skin near the tip was not high enough to treat dilated vessels [31]. Thus, several factors such as the light source, the coating material, as well as the coating location should be considered to achieve a higher temperature distribution near the tips, which would result in the realization of the practical use of the OMLA for selective photothermolysis.

## 5. Conclusions

In this work, we proposed and developed a gold-coated optical microneedle–lens array (OMLA) that has MNs and a lens array combined in a unit patch to accumulate introduced light at the tip of the microneedles for treatment targeting specific areas of skin, especially the reticular dermis layer.

Via the ray tracing simulation results, the dimensions of the microneedles and microlens were investigated. Based on the simulation results, we proposed a novel fabrication method for OMLAs, a frame-guided micromolding method. The method realized the fabrication of the OMLA including the assembly of two negative molds with alignment.

Next, a gold-coated OMLA was successfully fabricated by the gold deposition and modified lift-off method of a photoresist to expose the MN tips only. In the laser experiment, it was found that our developed OMLA device successfully accumulated light at the tips and blocked the transmission of light from other parts. In addition, quantitative analysis was performed to evaluate the power transmission efficiency by comparing the gold-coated OMLA, the OMLA without a coating, and a microneedle array without a lens array. It was found that light transmission of the gold-coated OMLA had 5.4-fold and 1.5-fold power increases compared with the MN array without a lens and the OMLA without a coating, respectively.

Finally, we investigated the temperature increase in the OMLA and its surroundings when the OMLA was irradiated with an IR laser. It was found that the peak temperature of the gold-coated OMLA at the tip of microneedles was 43 °C at the maximum when irradiated with a laser of 500 mW. Based on the heating evaluation, it is expected that several challenges regarding the temperature control and optimization of OMLA designs will be tackled for the further improvement of developed OMLAs as biomedical devices for selective photothermolysis.

## Data Availability

The original contributions presented in the study are included in the article, further inquiries can be directed to the corresponding author.

## Supplementary Materials

The following supporting information can be downloaded at https://www.mdpi.com/article/10.3390/mi15060725/s1. Table S1. Experimental parameters of three layers as a skin model that used in this work; Figure S1. Schematic diagram of OMLA design, including MNs and microlens; Figure S2. Simulation configuration overview for ray trajectory; Figure S3. COMSOL simulation results with various thicknesses: (a) 400 µm, (b) 800 µm, and (c) 1200 µm; Figure S4. Photons counted at the tip with different base layer thicknesses; Figure S5. Calculation results with respect to different incident angles: (a) relationship between the ratio of photons that reached the tip between incident angles, (b) incident angle = 1°, and (c) incident angle = 6°; Figure S6. Calculation results with respect to various misalignments: (a) relationship between the photon ratio and the misalignment distance, (b) misalignment distance = 90 µm, and (c) misalignment distance = 250 µm; Figure S7. Porcine skin surface after heating evaluation of OMLA; Figure S8. OMLA before/after heating evaluation; References [32,33,34,35,36,37] are cited in the supplementary materials.
