# Supplementary material for: Optical Microneedle–Lens Array for Selective Photothermolysis
Source: Micromachines (Basel). 2024 May 30;15(6):725. doi: 10.3390/mi15060725 (PMC11206131; doi:10.3390/mi15060725)
Supplement: Supplementary file 1 [file micromachines-15-00725-s001.zip › micromachines-3022257-supplementary.pdf]

micromachines

MDPI

Supplementary Materials: Optical Microneedle–Lens Array for Selective Photothermolysis

Jongho Park [1,†], Kotaro Shobayashi [2,†] and Beomjoon Kim [1,*]

[1] Institute of Industrial Science, The University of Tokyo, Tokyo 153-8505, Japan; johopark@iis.u-tokyo.ac.jp
[2] Department of Precision Engineering, School of Engineering, The University of Tokyo, Tokyo 113-8656, Japan; kotaro-s@iis.u-tokyo.ac.jp
* Correspondence: bjoonkim@iis.u-tokyo.ac.jp; Tel.: +81-3-5452-6224
† These authors contributed equally to this work.

S1. Modeling using the finite element method

We performed ray tracing evaluation by using the finite element method (FEM) on COMSOL software to investigate the optical path of photon that travels through microneedles (MNs) and microlens array. There are two ways to analyze optical characteristics: energetic particle and wave optical properties. Light waves are commonly used in optical coherence tomography (OCT), polarized light imaging. In our study, we focused on energetic particle analysis as our study aims to investigate the influence of photon interaction with tissue. For energetic particle analysis, Kulbelka-Munk (K-M) theory has been used to describe the characteristic of photons that propagate in forward and backward direction, perpendicular to the plane and to treat attenuation due to scattering and absorption. In order to reduce the amount of computation and simplify the equation, the theory assumes that all light propagates in all directions uniformly. The following equation of light propagation is used to describe light transport and can be expanded as a spherical harmonic equation.

$$\frac{1}{c}\frac{\partial}{\partial t}\Phi(r,t) = \nabla\left\{\frac{1}{3(\mu_s'+\mu_a)}\nabla\phi(r,t)\right\} - \mu_a\phi(r,t) + S(r,t) \quad \text{(S1)}$$

where, c is the light speed, r is the position vector, t is the time, $\phi(r,t)$ is the fluence value of radiant energy, $S(r,t)$ is the light intensity of an anisotropic light source, μ_s is the scattering coefficient, and μ_a is the absorption coefficient.

In this work, we used COMSOL software (version 5.6) to analyze the ray trajectory of each photon by using FEM. It is a general method for solving partial differential equation in two or three space variables. To solve the problem using this method, we divided the large system into smaller parts that are called finite elements. The simple equation that models these finite elements, is then assembled to final larger system to model the entire problem. FEM has an advantage that the boundary condition can be easily incorporated and thus it is commonly used for optical simulation for ray tracing each photon.

S1.1. Physical interaction of light and skin

The first interaction of light with tissue is explained by reflection and refraction. The angle of incident light and the refractive index of the medium determine the resulting direction of the light. As the index refraction of human skin is greater than the air, skin reflects 4–7% of the incident radiation, and the remaining 93–96% part penetrates inside the skin by Fresnel reflection [32]. Compared to the conventional light interaction with smooth surface, the consideration of the surface rugosity becomes more complex as scattering enters in the interaction. Scattering is the major cause of dispersion of large fraction of radiation emitted from the source. This effect can be one of the explanations about limited light penetration inside the skin. The scattering coefficient μ_s (mm^{-1}) depends on the wavelength.

On the other hand, light absorption is the transformation of light energy to heat. It is expressed by Lambert–Beer law,

$$I_t = I_0 * e^{-\mu_a * d} \quad \text{(S2)}$$

where, I_t (W/cm²) is the intensity of transmitted light, I_0 is the initial intensity, μ_a (mm⁻¹) is the absorption coefficient that depends on the wavelength, and l is the length of light path. Once the molecule absorbs the photonic energy, it will induce electronic excitation and generate heat. Absorption is the only way that light can interact with the tissue to induce thermal coagulation of the target. The human skin chromophores have a wide range of absorption bands from 200 to 1000 nm depending on their kinds as well as locations [32]. Among them, epidermal melanin that absorbs light from 300 to 1000 nm plays an important role in limiting the penetration of light in the skin.

The refractive index also plays an important role for the interaction of light and skin. Tom *et al.* irradiated the skin with various wavelengths and estimated the index of refraction of each layer [33]. It reported that each layer of a skin model has its own absorption and scattering coefficient as well as the index of refraction. In this work, three representative layers have been considered for our simulation: stratum cornea, epidermis, and dermis layer to simplify our simulation (Table S1).

Table S1. Experimental parameters of three layers as a skin model that used in this work [33,34].

Name of layer	μ_s (mm^{-1})	μ_a (mm^{-1})	l (thickness, μm)	Refractive index
Stratum cornea	100	0.9	20	1.52
Epidermis	45	2.6	200	1.34
Dermis	30	0.65	800	1.41

Finally, the boundary condition of light refraction can be simulated from Snell's law. When a photon interacts with another medium, the reflection probability (R) is calculated by:

$$R(\alpha) = \frac{(n_b - n_a)^2}{(n_b + n_a)^2} \quad \text{(S3)}$$

where, n_a and n_b are refractive indices of two different media *a* and *b*, respectively. When $\alpha > \sin^{-1}\frac{n_b}{n_a}$, the reflectance is: $R(\alpha) = 1$, in this situation, the light is totally reflected.

S1.2. Dimension of MNs and microlens array

In this section, we set experimental parameters for simulation based on predefined parameters such as physical dimension of the skin. The overview of our device is shown in Figure S1. First, we supposed that partial MN body will not be inserted completely due to the flexible characteristic of skin or the incomplete insertion. Thus, MNs that are not inserted was set to 200 μm [26]. The height of MNs has been set to 1 mm in order to make MNs reach near the reticular dermis. Moreover, this depth corresponds to the area where the treatment of a skin disease such as port wine stain remains difficult. In fact, it was reported that when the depth of lesion exceeds 1030 μm, the result of treatment is poor and complete blanching does not occur [25]. In case of more superficial lesions below than 830 μm, the response of treatment was reported to be much better [35]. In addition, this height also has an advantage: MNs less than 1 mm in height can significantly reduce the pain during the insertion [36]. The base of the MNs was calculated from the angle of the tip and the height of the MNs. The longer MN's pitch becomes, the easier MNs can be inserted. Thus, the pitch of MNs was set to 1.5 mm finally.

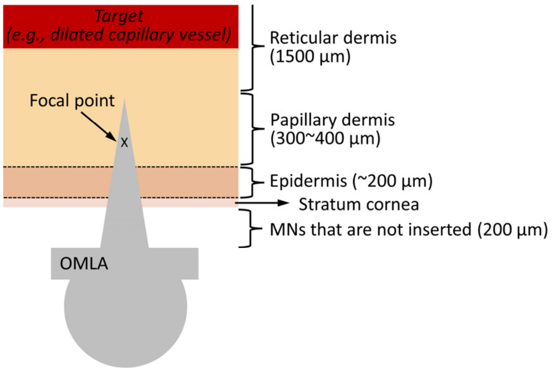

Figure S1. Schematic diagram of OMLA design, including MNs and microlens.

According to the microlens, parameters were chosen so that the numerical aperture can be maximized to gather a wide range of light. The high value of numerical aperture allows to capture more light compared to small numerical aperture.

The numerical aperture (NA) can be described as:

$$NA = \mathrm{n} \sin \vartheta \quad \text{(S4)}$$

where, n is the refractive index, ϑ is the maximum angle to the optical axis and the objective lens. By integrating the focal length f and the radius of the lens r_l, the formula can be re-written as:

$$NA = \frac{n * r_l}{\sqrt{r_l^2 + f^2}} \quad \text{(S5)}$$

Moreover, the focal length can be described with the radius curvature of the lens, $R_{curvature}$.

$$f = \frac{R_{curvature}}{\mathrm{n} - 1} \quad \text{(S6)}$$

By inserting this equation in NA, we can finally obtain:

$$NA = \frac{n(n-1)}{\sqrt{(n-1)^2 + \frac{R_{curvature}^2}{r_l^2}}} \quad \text{(S7)}$$

In order to maximize the NA, the radius of the lens and the radius curvature has been set to the same value as the minimum of radius curvature is the value of radius of lens. Thus, our lens shape is a plano-convex for the reason above. Finally, the focal point was set near to the tip of MNs so that we could ensure the accumulation of light at the tip. To do so, we took a radius of 1.5 mm so that we could have a focal length of 3 mm. Here, the thickness between MNs and microlens was set to 800 µm to satisfy the position of focal length.

S2. Simulation based on COMSOL application software

From the equation described above with the dimensions of MNs and microlens, we performed ray tracing simulation to analyze the trajectory of each photon in light propagation. First, one MN with one microlens, and following boundary condition had been set to bring the simulation closer to the reality. The MN inserted inside a skin model composed of three layers: stratum cornea, epidermis, and dermis. Each refraction index has been considered according to Table S1.

In order to block the transmission of photons which do not travel through the base of MNs, a wall was set to remove the unwanted photon as a boundary condition. In addition, the distance between a laser source and microlens was set to 200 µm. We supposed that this parameter does not influence the experience as the laser source propagates uniformly in one direction. And finally, the imaginary target (e.g. blood vessel) was placed at the tip of MNs so that it can accumulate and count the number of photons (Figure S2).

In this work, polylactic acid (PLA) was selected due to its transparency for optical application. From the ellipsometry measurements in previous literature [37], we can estimate that the index of refraction for PLA is from 1.448 to 1.499 with the light wavelength from 300 nm to 1300 nm. In addition, as we selected the wavelength to 595 nm, the corresponding index of refraction was 1.47 and used for simulations.

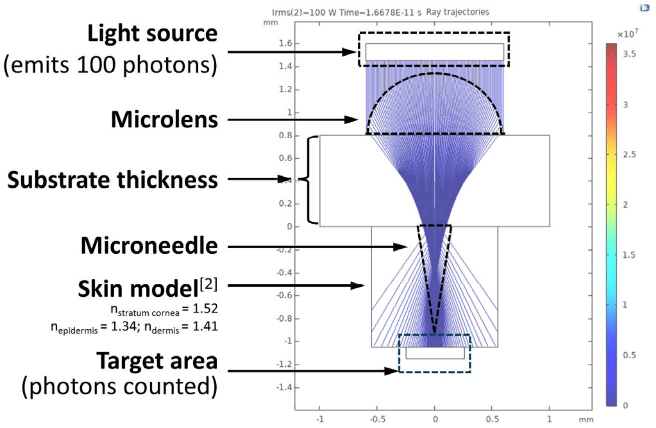

Figure S2. Simulation configuration overview for ray trajectory.

S2.1. Thickness control of the base layer in OMLA

In order to investigate the influence of the thickness of a base layer, we performed simulations to get photon counts that reached the tip with changing thickness of the base layer. From the simulation results, we confirmed that the light penetrates from the edge of the base layer when the thickness is 400 μm (Figure S3a). In this case, the light was absorbed by the melanin and then additional heat generation cannot be avoided, which cannot achieve our objective.

In case of thicker thickness, 1200 μm, the focal length was located at the base of MN. As shown in Figure S3c, photon was scattered at the base layer and thus light was scattered away along the MN, which does not satisfy the requirement of our objective, either. Thus, we calculated the number of photons arrived at the tip to find an optimal and maximum photon value by changing the thickness of a base layer. Finally, we confirmed that maximum photon numbers can be achieved when the thickness becomes 800, as shown in Figure S3b and Figure S4.

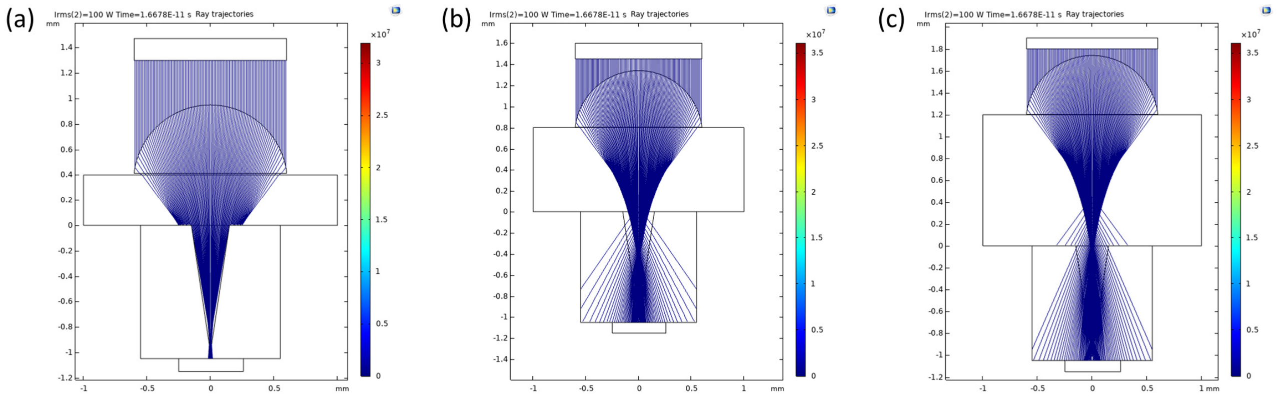

Figure S3. COMSOL simulation results with various thicknesses: (a) 400 μm, (b) 800 μm, and (c) 1200 μm.

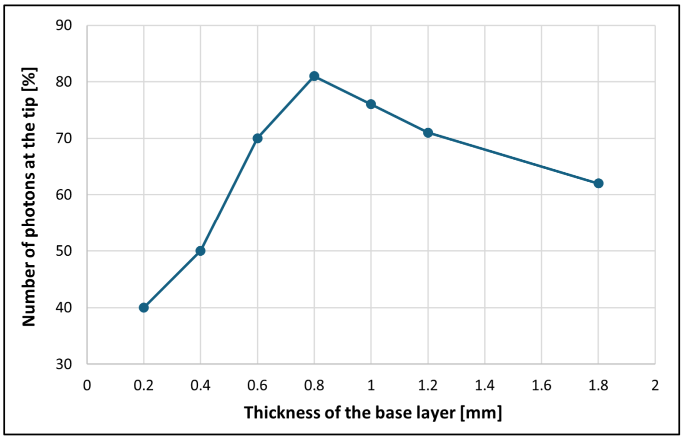

Figure S4. Photon counted at the tip with different base layer thicknesses.

S2.2. Influence of incidence angle

Next, we investigated the influence of light's incident angle. From the calculation result, we confirmed a gradual decrease of light transmission efficiency as the incident angle increases (Figure S5a). In addition, photon counts became almost zero at the incident angle over 10°, which means no more light was transmitted to the tip of OMLA with light scattering at the lower part of OMLA. Representative results with two different incident angles, 1 and 6°, are shown in Figure S5b and S5c, respectively. It was clearly observed that the incident light did not reach the tip with 6° of incident angle. From the result, we considered that the incident angle while using OMLA for treatment should be less 10° to get expected photothermal effect.

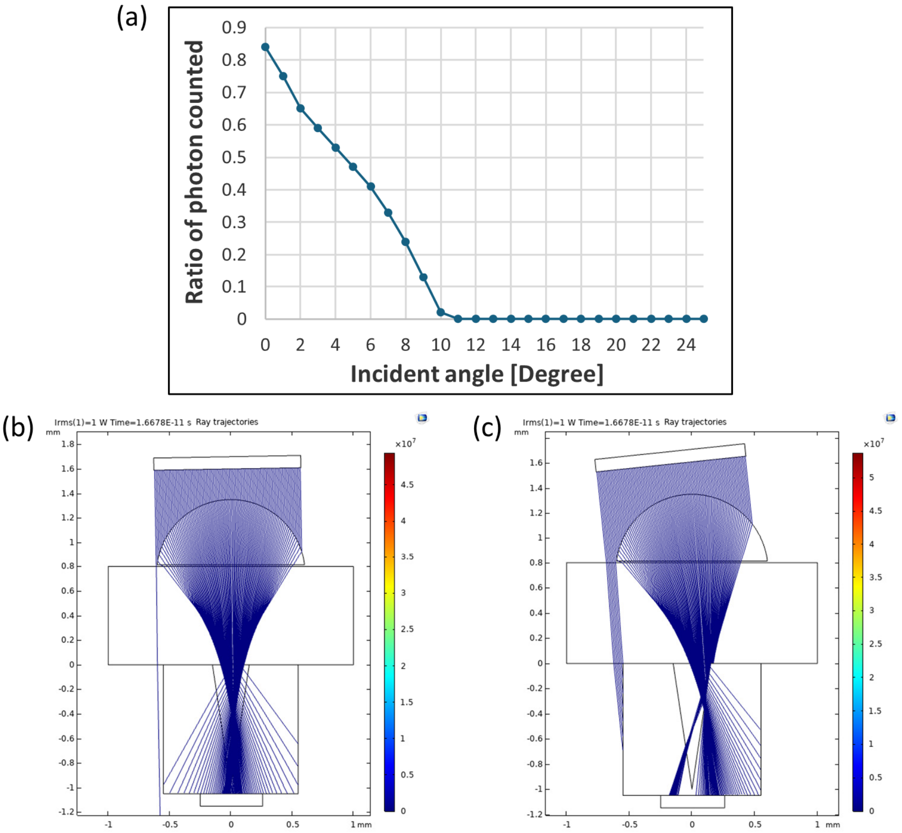

Figure S5. Calculation results with respect to different incident angles: (a) relationship between the ratio of photons that reached the tip between incident angles, (b) incident angle = 1°, and (c) incident angle = 6°.

S2.3. Influence of alignment between MNs and microlens

Finally, we investigated the influence of alignment between MNs and microlens. The z-axis reference for alignment was set in the center of microlens and MN base. Similar to the previous simulation, we counted the number of photons that arrived at the tip of MN and plotted the relationship between the ratio of light transmission and misalignment distance.

Figure S6a shows the relationship between misalignment distance and photon ratio. From the calculation result, we found that the tolerance for the misalignment would be around 100 μm in achieving at least 50% of transmitted photons. At the same time, it was suggested that the maximum tolerable misalignment was around 230 μm from simulation results. Figure S6b and S6c shows the simulation results with 90 and 250 μm of misalignment. With 90 μm of misalignment, we observed a light scattering at the lower part of MN, as shown in Figure S6b, due to its shifted position of focal length. Supposing that 90 μm misalignment would occur with the micromolding fabrication method, it would be necessary to consider the proper method to block the unexpected light transmission shown above. In addition, the misalignment distance exceeds 230 μm, it was observed from the simulation results that photons were no more transmitted at the tip of MNs as shown Figure S6c.

Considering the results so far, we figured out that the transmission efficiency of light in using OMLA would decrease by several factors described above. In addition, we expect that a supplementary measure would be necessary to maintain or increase the transmission efficiency as those factors are inevitable considering fabrication processes. As one of the possible solutions, we consider the coating of MN surfaces, which can block the scattering or escaping of light directly during its propagation. We finally chose gold as coating material as gold is considered to be feasible solution as it has sufficient reflectivity, biocompatibility, and processability with well-known microfabrication process [28].

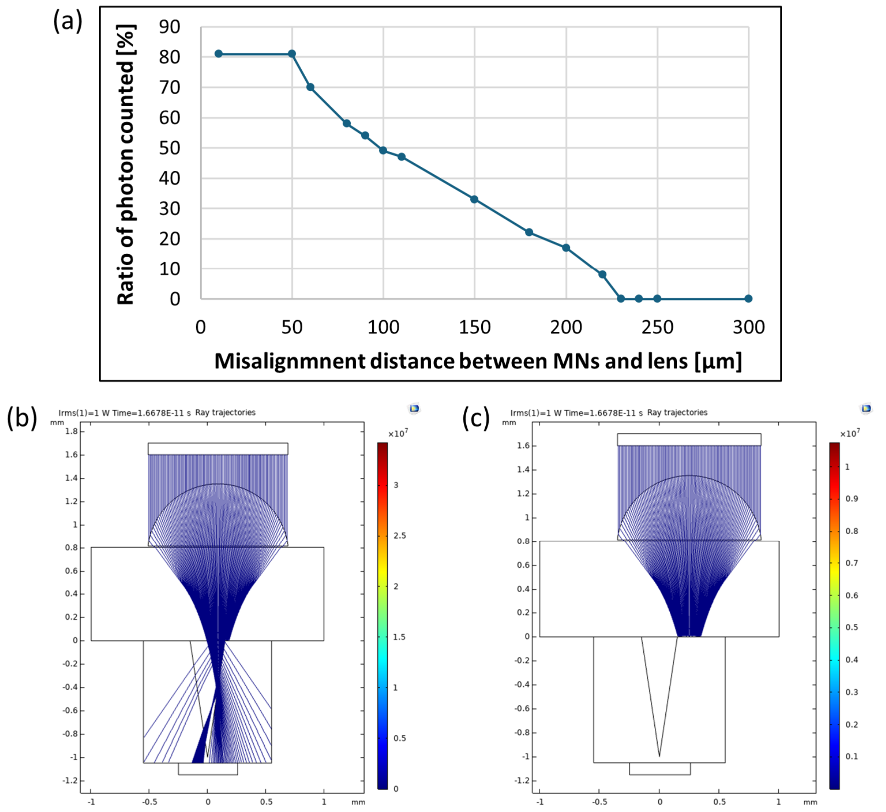

Figure S6. Calculation results with respect to various misalignments: (a) relationship between the photon ratio and misalignment distance, (b) misalignment distance = 90 μm, and (c) misalignment distance = 250 μm.

S3. Evaluation of MNs' skin insertion and the robustness of gold coating

We evaluated the skin insertion by OMLA as well as the robustness of gold layer coated onto OMLA after the evaluation of heating behavior. For the evaluation of skin insertion, we retrieved the porcine skin sample that was used for heating behaviors (4.4 Section, Main article). The skin was separated manually from the OMLA and was stained with 1% (w/v) of methylene blue solution for 15 min. After the dyeing process was finished, the solution was wiped with an ethanol-soaked wipe.

The result is shown in Figure S7. Red circles are pointing out the penetration marks that are formed by MNs. As it shows, we observed that some MNs could not successfully penetrate the skin surface. We considered that several factors caused the insufficient penetration: e.g. uneven and wet surface of a porcine skin, nonuniform force application, and horizontal motion during first punctuation. As the MNs of OMLA play an important role as a light transmission without being disturbed by surroundings, we considered that it is necessary to improve the skin penetration prior to *in vivo* evaluations such as animal experiments.

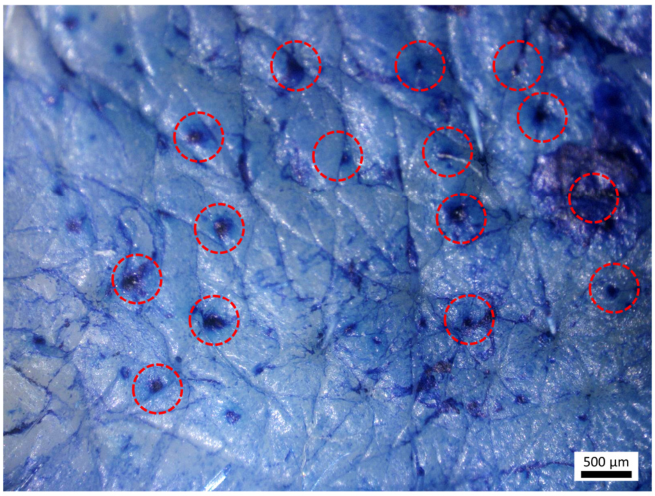

Figure S7. Porcine skin surface after heating evaluation of OMLA.

Next, we also evaluated the gold coating's robustness using OMLA after the previous evaluation. As gold plays an important role to make all light propagate toward the exposed MN tips, whether it can be maintained or not is important.

Figure S8 shows the microscopic images of used OMLA. Firstly, we confirmed that gold layers were intact even after a whole heating experiment. Thus, it was expected that our method for coating gold layer onto PLA microneedles was valid and feasible for further applications. Meanwhile, we also observed that several tips of MNs in OMLA were partially bent into a specific direction, which results in the application of one directional force. In addition, we obtained thermographic images showing MN's intact shapes (Figure 8, Main article), we expect that MNs were bent during the insertion onto a skin or the removal from the skin. As all OMLAs were applied onto a gelatin block as well as a porcine skin manually through this work, manual insertion can be one reason for the result. Along with the penetration issues described above, we consider that the establishment of the definite protocol for in vivo evaluation is also necessary to prevent the damage, e.g. bending, of MNs.

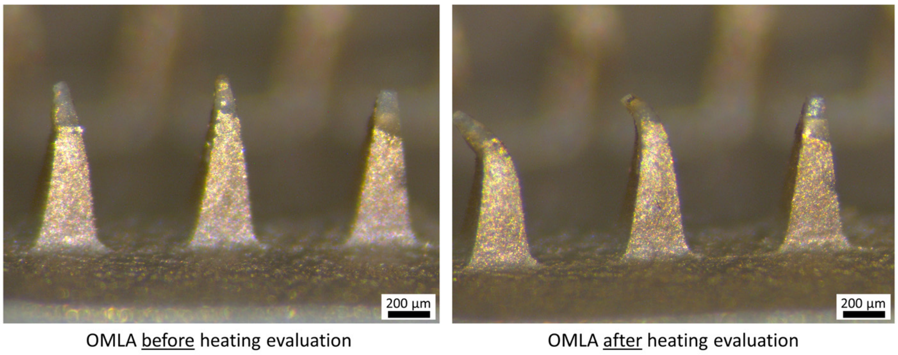

Figure S8. OMLA before/after heating evaluation.
